# Porcine Epidemic Diarrhea Virus and Its nsp14 Suppress ER Stress Induced GRP78

**DOI:** 10.3390/ijms24054936

**Published:** 2023-03-03

**Authors:** Wei Zeng, Jingping Ren, Gan Yang, Changsheng Jiang, Ling Dong, Qi Sun, Yaofang Hu, Wentao Li, Qigai He

**Affiliations:** 1 State Key Laboratory of Agricultural Microbiology, College of Veterinary Medicine, Huazhong Agricultural University, Wuhan 430070, China; aiyouwei@webmail.hzau.edu.cn (W.Z.); rjp@webmail.hzau.edu.cn (J.R.); yanggan@webmail.hzau.edu.cn (G.Y.); changshengjiang@webmail.hzau.edu.cn (C.J.); dong1001@webmail.hzau.edu.cn (L.D.); sunqi1019@webmail.hzau.edu.cn (Q.S.); hyf1997abc@webmail.hzau.edu.cn (Y.H.); wentao@mail.hzau.edu.cn (W.L.); 2 The Cooperative Innovation Center for Sustainable Pig Production, College of Veterinary Medicine, Huazhong Agricultural University, Wuhan 430070, China

**Keywords:** PEDV, ER stress, GRP78, nsp14

## Abstract

Porcine epidemic diarrhea virus (PEDV), a member of the α-coronavirus genus, can cause vomiting, diarrhea, and dehydration in piglets. Neonatal piglets infected with PEDV have a mortality rate as high as 100%. PEDV has caused substantial economic losses to the pork industry. Endoplasmic reticulum (ER) stress, which can alleviate the accumulation of unfolded or misfolded proteins in ER, involves in coronavirus infection. Previous studies have indicated that ER stress could inhibit the replication of human coronaviruses, and some human coronaviruses in turn could suppress ER stress-related factors. In this study, we demonstrated that PEDV could interact with ER stress. We determined that ER stress could potently inhibit the replication of GⅠ, GⅡ-a, and GⅡ-b PEDV strains. Moreover, we found that these PEDV strains can dampen the expression of the 78 kDa glucose-regulated protein (GRP78), an ER stress marker, while GRP78 overexpression showed antiviral activity against PEDV. Among different PEDV proteins, PEDV non-structural protein 14 (nsp14) was revealed to play an essential role in the inhibition of GRP78 by PEDV, and its guanine-N7-methyltransferase domain is necessary for this role. Further studies show that both PEDV and its nsp14 negatively regulated host translation, which could account for their inhibitory effects against GRP78. In addition, we found that PEDV nsp14 could inhibit the activity of GRP78 promotor, helping suppress GRP78 transcription. Our results reveal that PEDV possesses the potential to antagonize ER stress, and suggest that ER stress and PEDV nsp14 could be the targets for developing anti-PEDV drugs.

## 1. Introduction

Porcine epidemic diarrhea virus (PEDV) is an enveloped, positive-sense, single-stranded RNA virus belonging to the Alpha genus of the family Coronaviridae. The PEDV 5′-capped and 3′-polyadenylated RNA genome is approximately 28,000 nucleotides (nt) long and encodes two polyproteins (pp1a and pp1ab) which can be further cleaved into sixteen non-structural proteins (nsp1-nsp16) by viral proteases (nsp3 and nsp5), a hypothetical accessory protein (open reading frame 3, ORF3), and four structural proteins including spike protein (S), envelope protein (E), membrane protein (M), and nucleocapsid (N) [1,2]. PEDV is the etiological agent of porcine epidemic diarrhea (PED), a highly contagious disease characterized by vomiting, dehydration, anorexia, and watery diarrhea [3,4]. PED was first reported in the United Kingdom in the 1970s [5]. In 2010, PED caused by PEDV variant strains was recognized as a pandemic in several countries [4,6,7,8]. These variant strains are highly lethal to piglets and have serious adverse effects on the global pork industry [6,9,10,11]. PEDV strains can be divided into two genotypes, GI and GII. The GII genotype consists of several subtypes, among which the GII-a subtype and the GII-b subtype account for the major portion [12]. Most pandemic strains belonged to the GII genotype in the past decade [12,13,14].

The endoplasmic reticulum (ER) is a crucial organelle that coordinates the folding, assembly, and transport of nascent peptide chains [15]. High-quality and high-fidelity protein folding is critical for cellular function. However, protein folding is the most error-prone step in gene expression [16]. There are many mechanisms in the ER to ensure that proteins are folded correctly [16,17,18,19]. The homeostasis of the endoplasmic reticulum will be affected when the internal environment of the cell is disturbed, or the cell is subjected to external stimuli [20,21]. As a result, unfolded or misfolded proteins accumulate in ER lumen, inducing ER stress and activating the unfolded protein response (UPR) signaling pathways, including PRKR-like ER kinase (PERK)–eukaryotic translation initiation factor 2α (eIF2α), inositol-requiring protein 1α (IRE1α)–X-box-binding protein 1 (XBP1), and activating transcription factor 6 (ATF6) [22,23]. In the initial stage of ER stress, the UPR mainly acts to reduce protein synthesis and facilitate proper protein folding, helping reduce the accumulation of unfolded proteins and restore ER homeostasis. [24,25]. The PERK-eIF2α branch is activated to limit protein synthesis globally, reducing the protein folding load on the ER [26]. Additionally, PERK-eIF2α branch activation can also increase the translation of some ER stress-related factors, thereby facilitating protein transport within the ER lumen [27]. In addition, the IRE1α-XBP1 branch, which regulates genes involved in protein folding, trafficking, and degradation, will also be activated, helping to reduce the number of misfolded proteins [28]. Activation of ATF6 mainly upregulates the transcription of protein foldases and chaperones, such as glucose-regulated protein 78 (GRP78), GRP94, and ER protein 57 (ERp57), which play essential roles in enhancing ER folding capacity [29]. The activation of the UPR signaling pathways can help attenuate ER stress, facilitating cell survival [24]. However, once the UPR fails to restore ER homeostasis, prolonged ER stress will trigger apoptosis mediated by the PERK–eIF2α–ATF4–CHOP pathway [30].

The 78 kDa glucose-regulated protein (GRP78), which is also referred to as heavy chain binding protein (Bip) or heat-shock 70-kDa protein 5 (Hspa5), is a molecular chaperone that resides in the ER [31,32]. It belongs to the HSP70 family and shares 60% homology with other HSP70 family members [33]. There are two main domains in the structure of GRP78: ATP binding domain (ABD) at the amino-terminal and substrate binding domain (SBD) at the carboxyl-terminal [34,35]. GRP78 is a master regulator of ER homeostasis and stress response, involved in correcting protein folding and preventing the anterograde transport of unfolded or misfolded proteins [36,37]. Upregulation of GRP78 is often regarded as an ER stress activation marker [38,39,40]. Studies have reported that human coronaviruses could restrain this ER stress marker, while ER stress showed antiviral activity against them [41,42]. In this study, we demonstrated that PEDV could interact with ER stress. We revealed that ER stress is an effective host mechanism for restricting PEDV replication, and PEDV could significantly inhibit GRP78 at the protein level while GRP78 showed anti-PEDV activity. Among different PEDV proteins, we found that PEDV nsp14 played an essential role in the inhibition of GRP78 by PEDV and its guanine-N7-methyltransferase (N7-MTase) domain is necessary for this role. Moreover, our results indicate that both PEDV and its nsp14 can inhibit host translation, which could account for their inhibitory effects against GRP78 at the protein level. PEDV nsp14 can also dampen GRP78 at the transcription level by disrupting the activity of GRP78 promotor.

## 2. Results

### 2.1. ER Stress Inhibits PEDV Propagation

**Figure 1:**
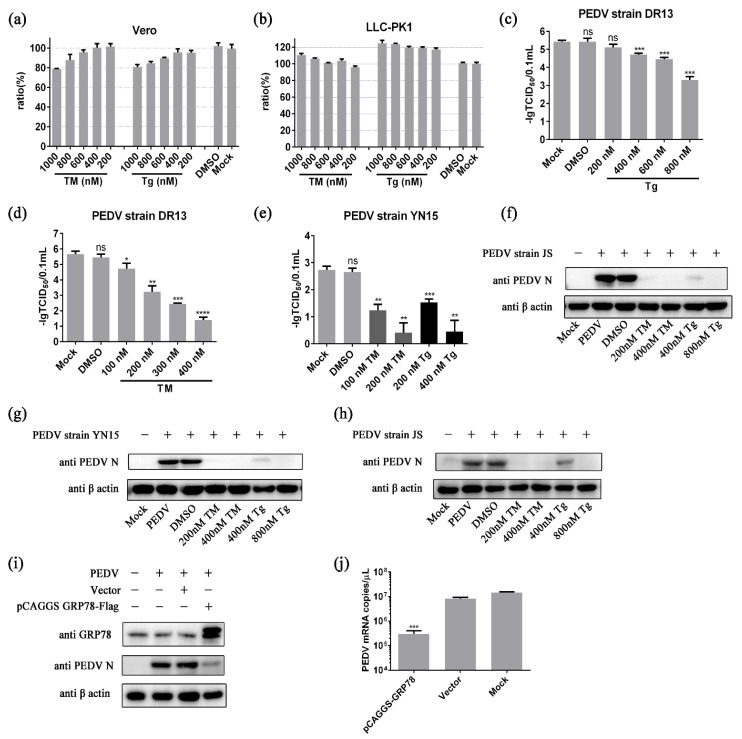
ER stress inhibits PEDV propagation. (**a**,**b**) Cell viability was measured by CCK8 assay at 48 h after treatment with TM or Tg. The cell viability of the mock group was used to normalize the results of other test groups. (**c**–**h**) Cells were pretreated with TM or Tg for 8 h before PEDV challenge (0.1 MOI). Samples were collected at 36 hpi or indicated time points. DMSO served as the treatment control. (**c**) The titer of PEDV strain DR13 determined in Tg pretreated Vero cells; (**d**) The titer of PEDV strain DR13 determined in TM pretreated Vero cells; (**e**) Viral titer determined in PEDV strain YN15 infected Vero cells; (**f**) The amount of PEDV N protein in PEDV strain JS infected Vero cells; (**g**) The amount of PEDV N protein in PEDV strain YN15 infected LLC-PK1 cells; (**h**) The amount of PEDV N protein in PEDV strain JS infected LLC-PK1 cells. β-actin was set as the loading control. (**i**,**j**) Vero cells were transfected with pCAGGS GRP78-flag or the control vector and exposed to 0.1 MOI PEDV 36 h after transfection. Samples were harvested at 36 hpi. The amount of PEDV was determined using western blot and RT-qPCR, respectively. “+” means that the corresponding material in the row has been added, and “−” means that the material is not added. Data represent the mean ± standard deviation (SD) of three independent experiments, and error bars represent the standard deviation. Asterisks indicate significant differences: * *p* < 0.05; ** *p* < 0.01; *** *p* < 0.001; **** *p* < 0.0001; ns, not significant.

Chemical endoplasmic reticulum stress has been shown to possess anti-coronavirus capabilities [41]. Two classical ER stress inducers, tunicamycin (TM) and thapsigargin (Tg), were applied to reveal the specific effect of ER stress on PEDV propagation [43,44]. Typically, cell viability below 80% is considered a threshold for cytotoxicity [45,46]. The results of Cell Counting kit-8 (CCK8) experiments showed that TM at concentrations below 400 nM and Tg at concentrations below 800 nM displayed no obvious cytotoxicity in Vero cells and LLC-PK1 cells (Figure 1a,b). These non-cytotoxic concentrations were applied in the subsequent experiments.

Cells were pretreated with TM or Tg for 8 h before PEDV challenge to rule out the direct effects of these chemicals on PEDV replication. The chemical ER stress potently inhibited PEDV strain DR13 replication in Vero cells in a dose-dependent manner. The viral titers of Tg treated groups steadily decreased from around 10^5.42^ TCID_50_/0.1 mL to 10^3.30^ TCID_50_/0.1 mL, with the doses of Tg increasing from 0 to 800 nM (Figure 1c). The same trend was observed in TM treated groups; the viral titers dropped sharply with the doses of TM increasing (Figure 1d). Experiments were also performed on PEDV strain JS and PEDV strain YN15, which belong to the GII-a sub-genotype and the GII-b sub-genotype, respectively. In experiments with PEDV strain YN15, the virus titer decreased with the concentration of inducers increasing (Figure 1e). The effect of ER stress on PEDV strain JS was determined by Western blot assay. The bands of PEDV N protein were almost invisible in the TM (200, 400 nM) treated groups and the Tg (400, 800 nM) treated group (Figure 1f). The influence of chemical ER stress on PEDV was also assessed in LLC-PK1 cells, a cell line from porcine kidney which was widely used in PEDV related research, to eliminate the impact of cell specificity [47,48]. The results in LLC-PK1 cells show the same trend as in Vero cells (Figure 1g,h). These results indicate that chemical ER stress could potently inhibit the replication of GII genotype PEDV strains.

Further study showed that the master regulator of ER stress, GRP78, possessed the potential to regulate PEDV replication. As shown in Figure 1i, the amount of PEDV N protein in the pCAGGS-GRP78-Flag transfection group was much lower than that in control groups, indicating that PEDV replication was suppressed in the GRP78 overexpression group. The results of quantitative PCR show the same trend. The mRNA copies in the GRP78 overexpression group were significantly less than in the control groups (Figure 1j).

### 2.2. PEDV Suppresses GRP78 Expression

**Figure 2:**
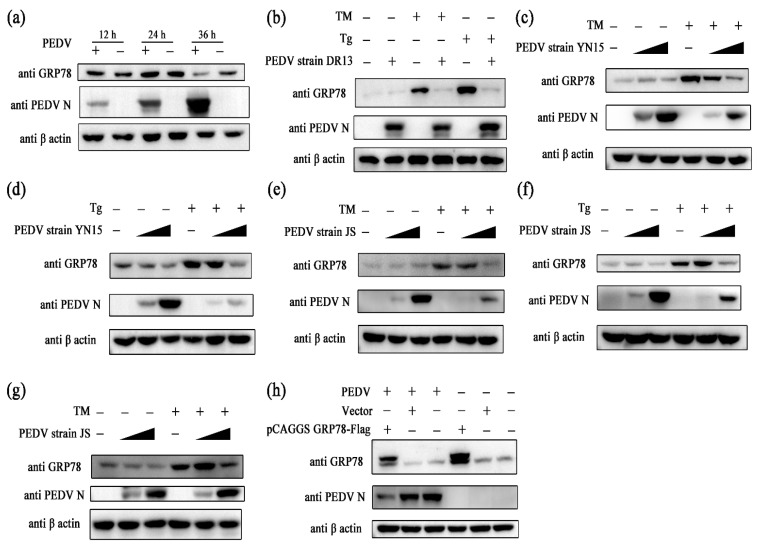
PEDV suppresses GRP78 expression. (**a**) Vero cells were challenged with 0.1 MOI PEDV. The amount of GRP78 protein and PEDV N protein were measured using Western blot; (**b**) Vero cells were first challenged with PEDV (0.1 MOI). TM (0.4 µM) or Tg (0.8 µM) was added to the culture medium at 24 hpi. Samples were harvested at 32 hpi and subjected to western blot analysis t; (**c**–**g**) Cells were first challenged with PEDV (0.01 MOI or 0.1 MOI). TM (0.4 µM) or Tg (0.8 µM) was added to the culture medium at 24 hpi. Samples were harvested at 32 hpi and subjected to western blot analysis; (**h**) Vero cells were transfected with pCAGGS GRP78-flag or the control vector and exposed to 0.1 MOI PEDV 36 h after transfection. Samples were harvested at 36 hpi and subjected to western blot analysis. β-actin was set as the loading control. “+” means that the corresponding material in the row has been added, and “−” means that the material is not added. Data represent the mean ± standard deviation (SD) of three independent experiments, and error bars represent the standard deviation.

To evaluate the effect of PEDV infection on ER stress, Vero cells were challenged with PEDV, and the samples were collected at different time points. The amount of GRP78 protein in the PEDV infected group was significantly less than in the control group at 36 hpi, indicating that PEDV may suppress GRP78 expression (Figure 2a). Further experiments were performed to confirm this hypothesis. Cells were exposed to PEDV first and then treated with ER stress inducers at 24 hpi. Samples were collected at 32 hpi. In Vero cells, it was observed that PEDV strain DR13 completely blocked TM- or Tg-induced GRP78 expression (Figure 2b). The GII genotype PEDV strains, PEDV strain YN15 and PEDV strain JS, also showed the capacity to suppress TM- or Tg-induced GRP78 expression (Figure 2c–f). In trials with LLC-PK1 cells, the inhibitory effect of PEDV on GRP78 remained (Figure 2g). To further verify the effect of PEDV on GRP78 expression, the eukaryotic expression vector of GRP78 (pCAGGS-GRP78-Flag) was applied. Among pCAGGS-GRP78-Flag transfection groups, the amount of GRP78 protein in cells exposed to PEDV was relatively less than in mock treated cells (Figure 2h). These results indicate that PEDV can inhibit GRP78 at the protein level.

### 2.3. PEDV nsp14 Inhibits GRP78

**Figure 3:**
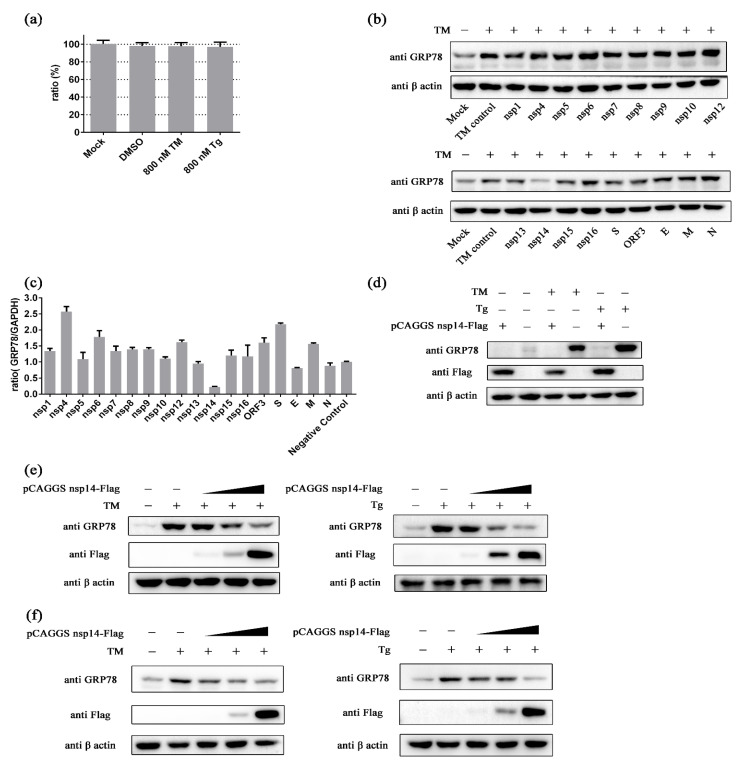
PEDV nsp14 inhibits GRP78. (**a**) The viability of HEK293t cells was measured by CCK8 assay at 48 h after treatment with TM or Tg. The cell viability of the mock group was used to normalize the results of other test groups. (**b**) The effect of different PEDV proteins on GRP78 at the protein level. HEK293t cells were transfected with plasmids and treated with 0.8 µM TM 36 h after transfection. Samples were harvested 44 h after transfection and subjected to western blot analysis; (**c**) The effect of different PEDV proteins on GRP78 at the transcription level. Samples were harvested 36 h after transfection and subjected to RT-qPCR analysis; (**d**,**e**) HEK293t cells were transfected with pCAGGS nsp14-Flag or the control vector and treated with TM (0.8 µM) or Tg (0.8 µM) 36 h after transfection. Samples were harvested 44 h after transfection and subjected to western blot analysis; (**f**) Vero cells were transfected with pCAGGS nsp14-Flag or the control vector and treated with TM (0.4 µM) or Tg (0.8 µM) 36 h after transfection. Samples were harvested 44 h after transfection and subjected to western blot analysis. β-actin was set as the loading control. Data represent the mean ± standard deviation (SD) of three independent experiments, and error bars represent the standard deviation. “+“ means that the corresponding material in the row has been added, and “−” means that the material is not added.

Eukaryotic expression vectors for different PEDV proteins based on the pCAGGS vector were constructed to explore which PEDV protein plays a crucial role in the inhibitory effect of PEDV on GRP78. The non-cytotoxic concentrations of ER stress inducers on HEK293t cells were determined first (Figure 3a). HEK293t cells were transfected with these vectors and treated with TM at 36 h after transfection. Samples were harvested at 8 h after adding TM. The results show that PEDV nsp14 could inhibit GRP78 at the protein level (Figure 3b). The influence of PEDV proteins on GRP78 at the transcription level was also evaluated. PEDV nsp14 exhibited the most robust repression ability against GRP78 at the transcription level (Figure 3c). We further confirmed the inhibitory activity of PEDV nsp14 against GRP78 at the protein level. In trials with HEK293t cells, either TM- or Tg-induced GRP78 upregulation can be inhibited by PEDV nsp14 (Figure 3d). This inhibitory effect showed a dose-dependent manner (Figure 3e). The impact of nsp14 on GRP78 was also evaluated in Vero cells. The results showed a similar trend as in HEK293t cells. The expression of GRP78 gradually decreased with the amount of nsp14 increasing in either TM or Tg treated groups (Figure 3f). These results indicate that PEDV nsp14 can inhibit GRP78 expression induced by chemical ER stress.

### 2.4. PEDV nsp14 N7-MTase Domain Is Crucial for Inhibiting GRP78

**Figure 4:**
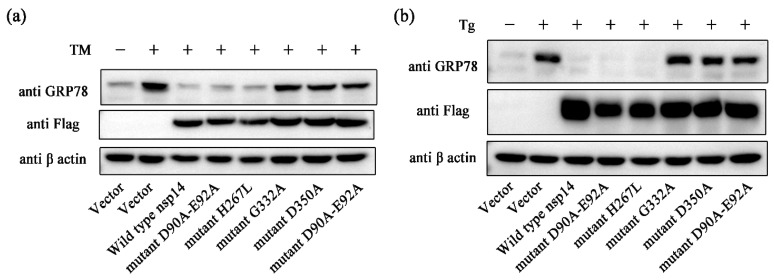
PEDV nsp14 N7-MTase domain is crucial for inhibiting GRP78. (**a**,**b**) Plasmids transfected HEK293t cells were treated with TM (0.4 µM) or Tg (0.8 µM), respectively. Western blot was performed to determine the expression level of proteins. β-actin was set as the loading control. “+” means that the corresponding material in the row has been added, and “−” means that the material is not added.

The non-structural protein 14 of coronavirus has two functional domains, in order from the amino terminus to the carboxyl terminus: 3′-to-5′ exoribonuclease (ExoN) and guanine-N7-methyltransferase (N7-MTase) domains [49,50]. Studies have illustrated that converting catalytic residues D_90_XE_92_ to alanine residues or converting H_267_ to leucine residue can silence the activity of the ExoN domain and converting Gly332 to alanine residue or converting Asp350 to alanine residue can abolish the activity of the N7-MTase domain [51,52,53]. We generated expression vectors encoding the catalytically inactive PEDV nsp14 mutants: mutant D90A-E92A, mutant H267L, mutant G332A, mutant D350A, and mutant H267L-D350A. The effect of these mutants on ER stress agonists induced GRP78 up-regulation was evaluated. Mutant D90A-E92A and mutant H267L can inhibit the expression of GRP78 as effectively as wild type nsp14 (Figure 4a). In contrast, mutant G332A, mutant D350A, and mutant H267L-D350A showed little restrictions on GRP78 up-regulation (Figure 4a). The same phenomenon was observed in the trial with Tg (Figure 4b). These results indicate that the N7-MTase domain plays a vital role in inhibiting the upregulation of GRP78.

### 2.5. PEDV and Its nsp14 inhibit GRP78 by Regulating Cellular Translation

**Figure 5:**
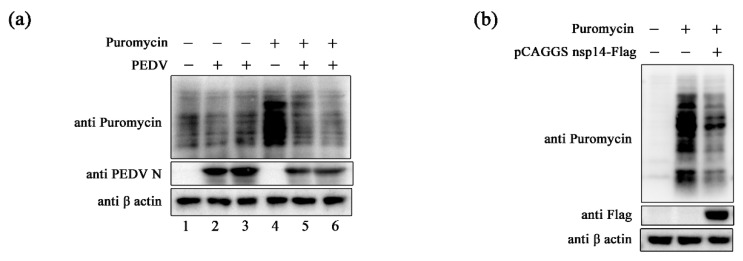
PEDV and its nsp14 inhibit cellular translation. (**a**) Vero cells were challenged with 0.1 MOI PEDV and subjected to puromycin incorporation assay at 36 hpi. Lanes 3 and 6 are repetitions of lanes 2 and 5, respectively; (**b**) HEK293t cells were transfected with pCAGGS nsp14-Flag or the control vector. Cells were subjected to puromycin incorporation assay 36 h after transfection. β-actin was set as the loading control. “+” means that the corresponding material in the row has been added, and “−” means that the material is not added.

It has been reported that human coronaviruses can induce translation shutdown globally [54]. Additionally, inhibition of host protein synthesis can help viruses evade host antiviral responses [54,55]. We tested whether PEDV possesses similar functions. Vero cells were challenged with PEDV first, and translation was evaluated by puromycin incorporation assay at 36 hpi. It was observed that PEDV infection could reduce Puro labeling (Figure 5a). In addition, translation in PEDV nsp14 overexpressing 293T cells was examined by puromycin incorporation assay to determine whether PEDV nsp14 exhibits a similar capacity. The amount of Puro-labeled proteins in the PEDV nsp14 overexpression group was much less than that in the control group (Figure 5b). These indicate that PEDV and its nsp14 can inhibit host translation, which may contribute to suppressing GRP78.

### 2.6. PEDV nsp14 Subcellular Localization

**Figure 6:**
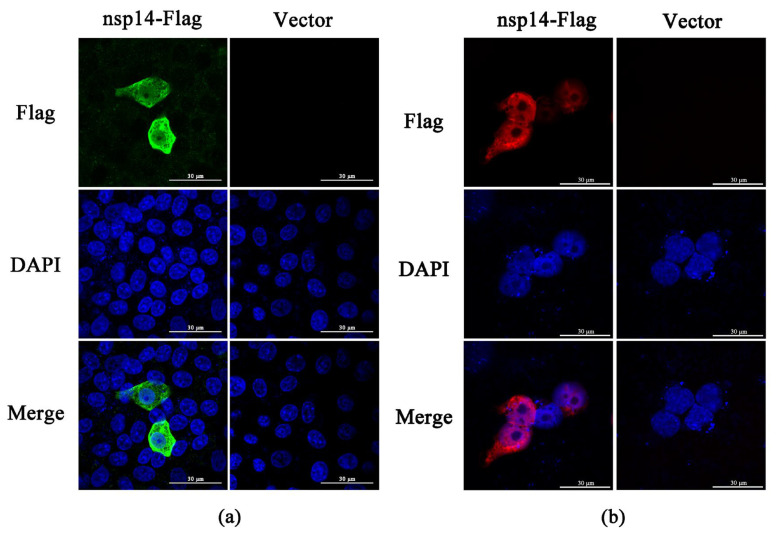
PEDV nsp14 subcellular localization. (**a**,**b**) Vero cells and HEK293t cells were transfected with pCAGGS nsp14-Flag or the control vector. Samples were harvested 36 h after transfection and subjected to IFA analysis. Images were generated using a confocal laser scanning microscope. Scale bar is 30 µm.

Subcellular localization of PEDV nsp14 was determined by a confocal laser scanning microscope. In Vero cells, PEDV nsp14 was distributed throughout the cell, including the nucleus (Figure 6a). In HEK293t cells, most of the PEDV nsp14 is located in the nucleus, while a small fraction of nsp14 can be detected in the cytoplasm (Figure 6b).

### 2.7. PEDV nsp14 Inhibits the Activity of GRP78 Promoter

**Figure 7:**
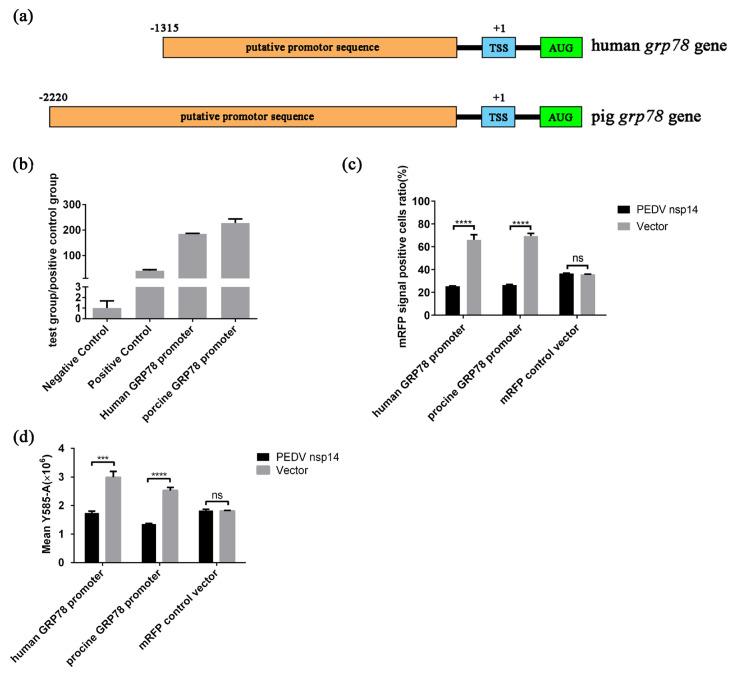
PEDV nsp14 inhibits the activity of GRP78 promoter. (**a**) Putative sequences for the GRP78 promoter were predicted using Promoter 2.0 Prediction Server; (**b**) Predicted sequences possess positive promoter activity. HEK293t cells were transfected with plasmids and subjected to luciferase reporter assay 36 h after transfection; (**c**,**d**) The pCAGGS nsp14-Flag or the control vector was co-transfected with the human-GRP78-promoter-mRFP, the porcine-GRP78-promoter-mRFP, and the mRFP control vector, respectively. Samples were harvested 36 h after transfection. Flow cytometry was performed to analyze mRFP signal positive cells ratio and the mean fluorescence intensity of mRFP positive cells. Data represent the mean ± standard deviation (SD) of three independent experiments, and error bars represent the standard deviation. Asterisks indicate significant differences: *** *p* < 0.001; **** *p* < 0.0001; ns, not significant.

To determine the influence of PEDV nsp14 on the GRP78 promoter, we predicated the human GRP78 promoter and the porcine GRP78 promoter (Figure 7a). Then, they were inserted into psicheckⅡ to replace the original promoter sequence upstream of the firefly luciferase coding sequence. The results indicate that the cloned sequences had vigorous transcriptional activity (Figure 7b). In subsequent experiments, we found that PEDV nsp14 could repress the expression of firefly luciferase mediated by the positive control vector (unmodified psicheckⅡ), which prevented us from judging whether nsp14 affects the activity of the promoter or acts on other processes of firefly luciferase expression. We, therefore, inserted these promoters into the pCAGGS-mRFP vector to replace the original promoter, constructing two new vectors, human-GRP78-promoter-mRFP and porcine-GRP78-promoter-mRFP. The human-GRP78-promoter-mRFP, the porcine-GRP78-promoter-mRFP, and the mRFP control vector (unmodified pCAGGS-mRFP) were, respectively, co-transfected into HEK293t cells with pCAGGS-PEDV-nsp14-flag or control vector (unmodified pCAGGS-flag) to evaluate the influence of PEDV nsp14 on the function of GRP78 promoters. The samples were harvested at 36 h after transfection, and the mRFP signals were analyzed by flow cytometry. In experiments using human-GRP78-promoter-mRFP or porcine-GRP78-promoter-mRFP, co-transfection with pCAGGS-PEDV nsp14-flag significantly reduced the mRFP signal positive cells ratio, and the mean fluorescence intensity of mRFP positive cells in PEDV nsp14 co-transfection groups was also relatively lower than that in control groups (Figure 7c,d). In addition, PEDV nsp14 could not affect the expression of mRFP mediated by the mRFP control vector (Figure 7c,d). These results indicate that PEDV nsp14 can negatively regulate the activity of both human GRP78 promoter and porcine GRP78 promoter, which could contribute to the inhibitory activity of PEDV nsp14 on the transcription of GRP78.

## 3. Discussion

ER stress, which plays an essential part in maintaining ER homeostasis and determining the fate of cells, possesses the potential to defend against coronavirus infection [24,30,56]. Chemical ER stress has been indicated to restrain the replication of human coronavirus-229E (HCoV-229E), Middle East respiratory syndrome coronavirus (MERS-CoV), and severe acute respiratory syndrome coronavirus 2 (SARS-CoV-2) [41]. In terms of porcine coronaviruses, it was found that inducing ER stress can effectively inhibit transmissible gastroenteritis virus (TGEV) propagation [57]. This study confirmed that both TM and Tg, the classical ER stress inducers, could suppress PEDV propagation. Since we pretreated cells with TM or Tg before PEDV challenge and replaced the supernatant with PEDV culture medium at 0 hpi during the experiment, the possibility that TM and Tg acted directly on PEDV can be ruled out. TM and Tg participate in different cellular activities, and activation of ER stress is the common denominator of their functions. TM activates ER stress via blocking N-terminal glycosylation, while Tg triggers ER stress through silencing Ca^2+^-dependent ER chaperone proteins [58,59]. Therefore, it is rational to consider that inducing ER stress accounts for the anti-PEDV activities of TM and Tg. In addition, three PEDV strains were applied in our tests. PEDV strain JS and YN15, belonging to the GII-a sub-genotype and the GII-b sub-genotype, respectively, represent current epidemic strains, while the GI genotype strain, DR13, represents classic strains [12,13,14]. ER stress showed inhibitory effects on PEDV strain DR13, strain YN15, and strain JS, indicating that ER stress could suppress the replication of PEDV strains belonging to the major genotypes. These suggest that ER stress is an effective anti-PEDV mechanism and could be a promising target for developing anti-PEDV drugs.

GRP78 is a protein with multiple functions. In most cases, GRP78 exists as a molecular chaperone in the ER [31,32]. It can regulate all the UPR pathways via interacting with their sensors [60]. GRP78 can also facilitate ER-associated degradation (ERAD) in the endoplasmic reticulum, thereby reducing misfolded proteins [61]. In addition, a fraction of GRP78 proteins can translocate to the cell surface and work as a multifunctional receptor, when the amount of GRP78 increases significantly [33]. Studies have shown that GRP78 could regulate virus infection. GRP78 is an essential receptor for several flaviviridae viruses, including Japanese Encephalitis Virus (JEV), Dengue virus (DENV), and Zika virus (ZIKV) [62,63,64]. In terms of coronavirus, GRP78 has been indicated to assist in MERS-CoV and SARS-CoV-2 invasion [65,66]. In this study, we found that GRP78 can suppress PEDV replication, which is consistent with the influence of ER stress on PEDV. Given that GRP78 is the classical marker of ER stress and the amount of GRP78 protein significantly increases during ER stress, it is possible that GRP78 played an essential part in the inhibition of PEDV by ER stress [38,39,40].

Virus infection is usually associated with ER stress. Hepatitis C virus was reported to induce autophagy by triggering ER stress, which could benefit its replication [67]. Zika virus can impair ER-stress-driven apoptosis while delaying apoptosis can help its propagation [68]. Japanese encephalitis virus and dengue viruses can enhance protein folding abilities by triggering ER stress [69]. Coronaviruses also regulate ER stress. Murine hepatitis virus (MHV) and severe acute respiratory syndrome coronavirus (SARS-CoV) can upregulate ER stress-related genes such as GRP78 and GRP94 at the transcription level [70,71]. Infectious bronchitis virus (IBV) can modulate apoptosis by triggering ER stress [72]. TGEV, belonging to the same genus as PEDV, was also indicated to induce ER stress in vitro and in vivo [57]. However, activating ER stress is not a fixed pattern for all coronaviruses. A recent study documented that HCoV-229E, MERS-CoV, and SARS-CoV-2 can downregulate the ER chaperone GRP78 and IRE1α [41]. In this study, we found that PEDV infection inhibited GRP78 expression. The results show that both TM- and Tg-induced GRP78 upregulation could be disrupted by PEDV. Moreover, PEDV could also suppress the overexpression of GRP78 by transfection. Given that GRP78 could inhibit PEDV replication and may play a part in the inhibitory effect of ER stress against PEDV, we speculated that PEDV may escape from the inhibitory effect of ER stress by inhibiting ER-stress-related factors, such as GRP78.

Studies have pointed out that coronavirus nsp14 can participate in regulating host responses. For example, SARS-CoV2 nsp14 can potently restrict interferon production, inhibit IRF3 nuclear localization, and prevent IFN-dependent ISG induction [54,73]. IBV nsp14 can antagonize the host antiviral response by inhibiting JAK-STAT signaling pathway [74]. Moreover, MHV nsp14 has been indicated to block host innate antiviral responses [75,76]. In this study, we found that PEDV nsp14 could inhibit GRP78, the master regulator of ER stress. PEDV nsp14 can suppress TM- and Tg-induced GRP78 expression in both HEK293t cells and Vero cells in a dose-dependent manner. This indicates that nsp14 plays an integral part in the anti-GRP78 effect of PEDV and may help antagonize the impact of GRP78 and ER stress on PEDV replication. 

Coronavirus nsp14 is a bifunctional protein containing an ExoN domain and an N7-MTase domain [49,50]. The primary function of the ExoN domain is to ensure the high-fidelity replication of the viral genome by detecting and removing mis-incorporated nucleotides, while the main function of the N7-MTase domain is to protect viral RNA from degrading by the cellular 5′-to-3′ exoribonucleases through participating in capping viral RNA [49,53,77]. In this study, we found that only the N7-MTase activity of PEDV nsp14 is required to suppress GRP78. According to a previous study, the N7-MTase domain of PEDV nsp14 is also involved in limiting IFN response [53]. This finding and our observations suggest that the N7-MTase domain of PEDV nsp14 functions to antagonize the host antiviral response in addition to capping viral RNA.

Human coronaviruses have been reported to evade host antiviral response by inhibiting host translation [54,55]. Several coronavirus non-structural proteins have been shown to be involved in regulating host translation. Severe acute respiratory syndrome coronavirus 2 (SARS-CoV-2) nsp1 can induce a shutdown of host protein production by interfering with the cellular translation machinery [55]. The nsp14 of several human coronaviruses can also suppress host translation [54]. In this study, we confirmed that PEDV possesses the capacity to restrict host translation and its nsp14 could also repress host translation, indicating that PEDV nsp14 may play an essential role in the inhibition of host translation by PEDV. Given that both PEDV and its nsp14 could down-regulate GRP78 at the protein level, we hypothesize that PEDV nsp14 could be a key factor bridging PEDV induced translation shutdown and its inhibitory effect on GRP78 at the protein level. However, the underlying molecular mechanism remains to be investigated.

We observed that PEDV nsp14 was distributed in the nucleus area in HEK293t cells and Vero cells. The nucleus is where eukaryotic transcription takes place [78]. Entering the nucleus is an essential character for transcription regulators [79]. Therefore, we speculate that the nucleus localization of PEDV nsp14 might be related to its ability to restrict GRP78 transcription. The gene promotor is a common target for transcription regulators. Some proteins have been described to enter the nucleus to trans-regulate the activity of gene promotors, such as the nuclear factor kappa B (NF-κB) protein, which modulates cellular responses by binding to gene promoters in the nucleus [80]. Viral proteins have similar potential. For instance, the core protein (HBc) of hepatitis B virus (HBV) was revealed to disrupt host gene expression via binding to gene promoters [81]. Our further results indicate that PEDV nsp14 can negatively regulate the activity of the GRP78 promoter. However, whether nsp14 achieves this function by binding to the promoter or influencing the corresponding trans-acting factors remains to be determined. 

In summary, we confirmed that ER stress could potently inhibit PEDV replication, and its marker protein, GRP78, played an essential role in this effect. Furthermore, we revealed that PEDV infection could negatively regulate GRP78 expression, and the N7-MTase domain of PEDV nsp14 played a part in this inhibitory effect. Both PEDV and its nsp14 could interfere with host translation which might account for the down-regulation of GRP78 at the protein level. Moreover, PEDV nsp14 could also repress GRP78 at the transcription level by disrupting the activity of the corresponding promoter. This study demonstrated for the first time that PEDV can antagonize ER stress-related factors. In addition, our observations on PEDV nsp14, combined with its important role in viral replication, suggest that PEDV nsp14 could be a good target for anti-PEDV drug development [49,50,53].

## 4. Materials and Methods

### 4.1. Viruses, Cells, Reagents, and Consumables

Vero cells (CCL81), LLC-PK1 cells, and HEK293t cells were cultured in Dulbecco’s Modified Eagle Medium (DMEM) (Gibco, Waltham, MA, USA) supplemented with 10% FBS (NEWZERUM, Christchurch, New Zealand) and incubated at 37 °C with 5% CO_2_. PEDV strain YN15 (GenBank accession No. KT021228.1) and PEDV strain JS (Genomic information has been uploaded to GenBank, but is currently under wraps) were cultured in DMEM containing trypsin (Gibco, Waltham, MA, USA) at a dose of 8 µg/mL. PEDV strain DR13 (GenBank accession No. JQ023161) was cultured in DMEM. Mouse anti-PEDV S protein monoclonal antibody and mouse anti-PEDV N protein monoclonal antibody were generated in our laboratory [82]. Rabbit anti-GRP78 polyclonal antibody, Rabbit anti-β-Actin Monoclonal antibody, Rabbit anti-flag polyclonal antibody, HRP goat anti-mouse IgG, and HRP goat anti-rabbit IgG were purchased from Proteintech (Wuhan, China). Alexa Fluor 488 Donkey anti Mouse IgG, and Alexa Fluor 594 Donkey anti Rabbit IgG were purchased from AntGene (Wuhan, China). Mouse anti-Puromycin monoclonal antibody (Anti-Puromycin Antibody, clone 12D10 ZooMAb^®^ Mouse Monoclonal) were purchased from Sigma-Aldrich (Darmstadt, Germany). Tunicamycin (TM), thapsigargin (Tg), MG132, and 3-Methyladenine (3-MA) were purchased from MedChemExpress (Shanghai, China). Cell culture consumables (flasks, plates, etc.) were purchased from NEST (Wuxi, China)

### 4.2. Cell Counting kit-8 Assay

Cell viability was measured through Cell counting kit-8 (CCK8) (Beyotime, Shanghai, China). Cells were cultured to 100% confluency in 96-well plates. After washing with PBS 3 times, cells were incubated with DMEM containing chemicals for 48 h. Then, cells were washed with PBS 3 times and incubated with DMEM containing 9.09% CCK-8 reagent for 2 h in the dark. After a slight shake, the optical density 450 of each well was measured through a Multimode Plate Reader (VICTOR Nivo, PerkinElmer, Waltham, MA, USA). This equation calculated cell viability ratio: cell viability ratio = (As − Ab) ÷ (Ac − Ab) × 100%. As: OD450 values of chemicals treated wells; Ab: OD450 values of wells without cells; Ac: OD450 values of mock-treated wells.

### 4.3. TCID_50_ Assay

After being lysed by 3 freeze/thaw cycles, samples were centrifuged at 12,000× *g* for 10 min at 4 °C. Eight serial ten-fold dilutions of the sample supernatant were made in DMEM. Cells were cultured to 100% confluency in 96-well plates. After washing with PBS 3 times, cells were incubated with different dilutions for 72 h. TCID_50_ value of each sample was calculated according to the Reed–Muench method established by L. J. Reed and H. Muench.

### 4.4. Transfection

Cells were seeded in plates and cultured until they reached approximately 80% confluence. Then, cells were transfected with Plasmids using Lipo8000 transfection reagent (Beyotime, Shanghai, China) according to the manufacturer’s instructions. 

### 4.5. Indirect Immunofluorescence Assay

Cells were permeabilized with 0.1% Triton X-100 (dissolved in PBS) for 10 min at room temperature after fixing with 200 µL 4% Paraformaldehyde (dissolved in PBS) for 20 min at room temperature. Then, cells were blocked with PBS solution containing 1% BSA and 0.1% Tween-20 for 2 h at room temperature. Next, cells were incubated with PBS containing 0.1% primary antibody for 60 min at 37 °C. After washing with PBS 3 times, cells were incubated with PBS containing 0.1% fluorescently labeled secondary antibody for 30 min at 37 °C. Then, cells could be subjected to DAPI (Beyotime, Shanghai, China) for 5 min if it is needed to stain nuclei. Finally, after washing with PBS three times, images were captured using an inverted fluorescence microscope (Eclipse TI-U, Nikon, Tokyo, Japan) or a confocal microscope (STORM, Nikon, Tokyo, Japan).

### 4.6. Western Blot

Cells were harvested in RIPA Lysis Buffer (Beyotime, Shanghai, China). SDS-PAGE Sample loading buffer (5×) (Biosharp, Beijing, China) was added to the homogenized samples. The supernatant of samples was loaded onto SDS-PAGE gels (Epizyme Biotech, Shanghai, China) and subjected to electrophoresis. Separated proteins were transferred onto polyvinyl difluoride (PVDF) membranes (Biosharp, Beijing, China). Membranes were blocked with 5% skim milk (diluting in TBS buffer containing 0.05% Tween-20, TBST) for 2 h at room temperature. Then, membranes were incubated in TBST containing 0.05% primary antibody for 2 h at room temperature. After washing with TBST 3 times, membranes were incubated in TBST containing 0.05% HRP goat anti-mouse or HRP goat anti-rabbit antibodies for 2 h at room temperature. The signal was developed by incubating membranes with ECL Chemiluminescence substrate (Vazyme, Nanjing, China). The images were captured using a chemiluminescence detector (Tanon5200, Tanon, Shanghai, China).

### 4.7. Reverse-Transcription Quantitative PCR

RNA of samples was extracted using the Simply P Total RNA Extraction kit (Bioflux, Hangzhou, China). The corresponding cDNA was generated using HiScript II Q RT SuperMix For qPCR (Vazyme, Nanjing, China). SYBR green based quantitative PCR was performed by using Hieff UNICON qPCR SYBR Green Master Mix (Yeasen, Shanghai, China), and TaqMan based quantitative PCR was performed by using Hieff UNICON qPCR TaqMan Probe Master Mix (Yeasen, Shanghai, China). All of the real-time PCR tests were performed on QuantStudio real-time PCR system (Applied Biosystems, Waltham, MA, USA). Primers and a probe used in this study are listed in Table 1.

### 4.8. Puromycin Incorporation Assay

Cells challenged with PEDV or transfected with plasmids for an indicated time were incubated with DMEM containing 10% FBS and 20 μM puromycin for 15 min. Samples were then harvested using Laemmli sample buffer containing 2-mercaptoethanol. Western blot was performed to evaluate Puro labeling.

### 4.9. Flow Cytometry

Cells were dissociated into single-cell suspension by trypsinization 48 h after transfection. More than 10,000 live cells were measured in each sample by using the Cytoflex-LX flow cytometer (Beckman Coulter, CA, USA). 

### 4.10. Luciferase Reporter Assay

Luciferase activity was measured using the Dual Luciferase Reporter Gene Assay Kit (Yeasen, Shanghai, China). The firefly luciferase activity was normalized to the Renilla luciferase activity (firefly luciferase/Renilla luciferase), and expression is presented as relative luciferase activity.

### 4.11. Significant Difference Analysis

Differences between the two groups were analyzed by using Student’s *t*-test. Asterisks indicate significant differences: * *p* < 0.05; ** *p* < 0.01; *** *p* < 0.001; **** *p* < 0.0001; ns, not significant.

## Figures and Tables

**Table 1:** Primers and a probe used in this study.

Primer	Sequence (5′–3′)
PEDV F	CGTACAGGTAAGTCAATTAC
PEDV R	GATGAAGCATTGACTGAA
PEDV probe-M	FAM-TTCGTCACAGTCGCCAAGG-TAMRA
Human-GRP78-F	CATCAACGAGCCTACGGCA
Human-GRP78-R	AGACACATCGAAGGTTCCGC
Human-GAPDH-F	CACCATCTTCCAGGAGCGA
Human-GAPDH-R	ATGACGAACATGGGGGCATC

## Data Availability

Data sharing not applicable.
